# Multifaceted Assessment of Quality of Life in Hospitalized Adolescents Aged 11–18 with Cardiological Problems

**DOI:** 10.3390/children12121661

**Published:** 2025-12-08

**Authors:** Agnieszka Pluta, Alicja Marzec, Monika Chojnowska, Mariola Głowacka

**Affiliations:** 1 Division of Community Nursing, Faculty of Health Sciences, Ludwik Rydygier Collegium Medicum in Bydgoszcz, Nicolaus Copernicus University in Toruń, ul. Łukasiewicza 1, 85-821 Bydgoszcz, Poland; 2 Independent Public Healthcare Institutions in Żuromin, 09-300 Żuromin, Poland; 3 Collegium Medicum, The Mazovian University in Płock, 09-402 Płock, Poland

**Keywords:** quality of life, adolescents, cardiological problems, pediatric nursing, nursing interventions, KIDSCREEN-52

## Abstract

Background: Cardiological conditions in adolescents can impair health-related quality of life (HRQoL), influencing physical, emotional, and social functioning. Identifying sociodemographic and psychosocial determinants is essential for targeted multidisciplinary interventions involving pediatric cardiologists, nurses, and psychologists. This study assessed HRQoL in hospitalized adolescents with cardiologic problems. Methods: A cross-sectional study was conducted among 100 adolescents aged 11–18 years hospitalized in a pediatric cardiology ward in Poland (June–December 2022). HRQoL was measured using the validated Polish version of the KIDSCREEN-52 questionnaire. Data on demographics, family and financial situation, and pain were collected. Non-parametric tests and Spearman’s correlations were applied; *p* < 0.05 was considered significant. Results: The highest HRQoL scores were observed in Social Acceptance (mean 86.3 ± 17.9), while the lowest scores were found in School Environment (49.2 ± 21.4). Boys had significantly higher Physical Well-being and Self-perception scores than girls (*p* = 0.019, *p* = 0.031). Older age correlated negatively with Moods and Emotions (*r* = −0.216, *p* = 0.031) and Peer Relationships (*r* = −0.300, *p* = 0.002). Rural residence was associated with stronger family relationships (*p* = 0.025). A better financial status correlated with higher family relationship and financial resource scores. Pain was linked to poorer physical and emotional well-being. Conclusions: The health-related quality of life (HRQoL) of adolescents hospitalized for cardiac conditions is mainly affected by socio-demographic factors, such as gender, age, place of residence, perceived socioeconomic status, and experiences of pain and discomfort. Girls, older adolescents, urban residents, and those reporting poorer socioeconomic conditions and pain had lower HRQoL scores in specific areas. Conversely, family structure and the presence of chronic diseases did not significantly influence HRQoL outcomes.

## 1. Introduction

Cardiological conditions in children and adolescents have a profound impact on their health-related quality of life (HRQoL). These conditions influence not only physical ability but also emotional health and social participation, affecting not only physical functioning but also emotional well-being and social integration [1,2].

In Poland, these issues and conditions are increasingly recognized as a significant challenge in pediatric healthcare [3]. Managing chronic heart problems during adolescence is complex and requires a multidisciplinary approach, with nurses playing a crucial role by providing comprehensive, developmentally appropriate care [4].

A child with a cardiological condition is cared for by an interdisciplinary team consisting of a pediatrician, cardiologist, surgeon, nurse, psychologist, physiotherapist, and dietitian. Each member of this team plays a clearly defined role in addressing the diverse needs of adolescents with heart diseases.

Physicians not only coordinate the diagnostic and therapeutic processes but also play an important role in providing psychological support by delivering clear information, fostering a sense of safety, and strengthening the patient’s trust in the treatment process. A skilled and empathetic clinician provides reassurance that the chosen therapeutic pathway is both safe and likely to be effective. This sense of security is fundamental in the assessment of the quality of life of a chronically ill patient.

Pediatric nurses, together with other specialists, provide comprehensive support that includes pain management, psychological counseling, educational guidance, and interventions aimed at improving patients’ psychological, physical, and social well-being. Such empathetic, evidence-based, and emotionally supportive care significantly enhances adolescents’ ability to cope with illness and hospitalization [5,6].

A holistic, patient-centered nursing approach tailored to adolescents’ developmental stage can reduce the burden of illness and promote psychosocial adaptation [4,7,8]. The continuity of care is ensured by outpatient nurses working in cardiology clinics as well as school nurses operating within the educational environment. Nurses often act as advocates for educational and family needs, supporting academic adjustments and the reintegration of students into school and peer groups [9]. Previous international studies have consistently shown that adolescents with congenital heart disease (CHD) or other chronic cardiac conditions report lower HRQoL compared to their healthy peers. For example, Bratt and Moons [1] emphasized that HRQoL is not determined solely by disease severity, while Latal et al. [10] demonstrated long-term psychosocial challenges among children after open-heart surgery. Ferro and Boyle [11] confirmed that chronic illness is linked to a reduced self-concept, and Badawy et al. [12] demonstrated that psychosocial and organizational factors constitute significant barriers negatively affecting the quality of life of young patients with chronic illnesses. These challenges align with broader research indicating that chronic conditions during adolescence and young adulthood require substantial psychological and social adjustment [13,14]. Additionally, peer support is recognized as a protective factor for adolescents with chronic or rare conditions, helping improve coping and emotional functioning [15]. In the school setting, nurses also play a vital role in promoting educational inclusion and supporting children with chronic illnesses [9]. These findings suggest that HRQoL in adolescents with cardiological conditions is multidimensional, influenced by both medical and psychosocial factors.

In Poland, research has also explored the quality of life (QoL) among pediatric and cardiology patients. Mazur et al. [16] provided the Polish validation of the KIDSCREEN questionnaire, establishing normative data for children and adolescents. More recently, Świerczek [17] found that children after cardiac surgery often report postoperative symptoms, such as dyspnea (75.5%) and reduced exercise tolerance (66.7%), with QoL further impacted by socioeconomic status, scars, and repeated hospitalizations. Additionally, Piotrowicz-Wójcik et al. [9] showed decreased HRQoL, especially in emotional areas, among Polish children with hereditary angioedema, highlighting the need for systematic QoL assessment in pediatric patients with chronic or rare conditions. Earlier studies also emphasized the importance of psychosocial and functional factors: Szyguła-Jurkiewicz et al. [18] highlighted QoL as a vital indicator of health status and treatment effectiveness in cardiology.

Nevertheless, most of these studies were conducted among community or outpatient groups, or during long-term follow-up after surgery. Very few investigations have focused on hospitalized adolescents, even though hospitalization introduces unique stressors such as disruption of education, limited peer contact, and increased psychological distress [12,19]. To date, no research has systematically examined the HRQoL of hospitalized adolescents aged 11–18 with cardiovascular problems in Poland using a validated tool, such as the KIDSCREEN-52 [16,20].

Given these considerations, this study aimed to evaluate the HRQoL of hospitalized adolescents with cardiological conditions, taking into account the individual characteristics of the affected child, as well as sociodemographic characteristics, family, and school environment factors. The theoretical part of the study considered the role of the nurse in fulfilling caring, supportive, and coordinating roles in the therapeutic process for children with cardiac problems.

## 2. Methodology

The study population consisted of 100 adolescents aged 11–18 hospitalized due to cardiologic problems in the Department of Pediatrics and Cardiology at the Józef Brudziński Regional Children’s Hospital in Bydgoszcz. Patients and their legal representatives voluntarily provided informed, written consent to participate in the survey after being informed about the study’s purpose. The study was conducted between 21 June 2022, and 31 December 2022, after obtaining approval from the Bioethics Committee (KB 358/2022).

The study used the standardized KIDSCREEN-52 questionnaire and a custom survey questionnaire designed by the authors, which included two open-ended and six closed-ended questions regarding gender, age, place of residence, family and financial situation, chronic illnesses, and perceived pain.

The KIDSCREEN-52 questionnaire, in its validated Polish version [16], consists of 52 questions addressing aspects of physical health, mental health, social functioning, and material conditions. It encompasses 10 domains (dimensions) of quality of life. Only one dimension focuses on physical health. Three areas—psychological well-being, moods and emotions, and self-perception—relate to mental health. Five domains focus on social functioning: independence, relationships with parents and home life, peer contacts and social support, school environment, and social acceptance. The tenth dimension, financial resources, pertains to material conditions [16,20].

The KIDSCREEN-52 questionnaire in its Polish adaptation was developed and validated by Mazur et al. [16]. Validation studies confirmed the high validity and reliability of this tool in the population of children and adolescents aged 8–18 years. The internal consistency indices (Cronbach’s alpha) for the individual scales ranged from 0.77 to 0.89, confirming its good psychometric properties.

Inclusion and Exclusion Criteria: Inclusion criteria included a confirmed diagnosis of a cardiologic condition requiring hospitalization, written consent from the patient and their legal guardian, and cognitive ability to complete the questionnaire. Patients with intellectual disabilities or comorbid conditions that could significantly impact HRQoL assessments were excluded, including those under 11 years old and over 18 years old.

The study included 100 children hospitalized in the cardiology ward. All of the children presented with cardiac symptoms of varying severity. Some were undergoing diagnostic procedures, while others were attending periodic follow-up examinations after previously performed surgical treatment, e.g., due to congenital heart defects, where such monitoring is essential until the completion of the growth period. Within the study group, 33 children were diagnosed with an additional chronic non-cardiac condition alongside their cardiac disease. Among the cardiac conditions observed in the participants were: congenital heart defects (9 children), cardiac arrhythmias (4 children), and pulmonary arterial hypertension (1 child). The most common non-cardiac chronic conditions included: thyroid diseases (6 children), bronchial asthma (5 children), food and inhalant allergies (4 children), ulcerative colitis (2 children), celiac disease (1 child), and epilepsy (1 child).

Potential Bias and Limitations: One limitation of the study was the heterogeneity of the cardiac conditions within the sample, which could introduce variability in HRQoL outcomes. Additionally, the duration of hospitalization and previous medical interventions were not controlled variables, which could potentially affect HRQoL assessments.

### Statistical Analysis

Data were analyzed using Statistica 13 software. Mann–Whitney U tests were applied for group comparisons and Spearman’s correlation to examine relationships between HRQoL dimensions. Results with *p* < 0.05 were considered significant.

For the interpretation of results, we supplemented the analysis with the effect size for the Mann–Whitney U test, calculated as the *r* coefficient using the following formula

$$r=\frac{Z}{\sqrt{N}}$$

where *Z* is the test statistic and *N* is the total sample size.

- For the comparison of Physical Well-being between boys and girls (*Z* = −2.344; *p* = 0.019), the effect size coefficient was *r* = −0.234, which corresponds to a small-to-moderate effect.
- For the comparison of Self-perception (*Z* = −2.153; *p* = 0.031), the effect size was *r* = −0.215, also indicating a small-to-moderate effect.

## 3. Results

The sociodemographic characteristics of the study population are presented in Table 1.

Table 2 presents the reasons for hospitalization among the study participants.

The results of the KIDSCREEN-52 questionnaire, calculated according to the scoring key, allow for the determination of 10 indicators describing various aspects of children’s lives, including physical, emotional, environmental, and social dimensions. The highest mean score was observed in the “Social Acceptance” domain (86.3 ± 17.9), suggesting that adolescents felt generally accepted by their peers. In contrast, the “School Environment” domain had the lowest score (49.2 ± 21.4), highlighting considerable difficulties in academic functioning and indicating this area as the most critical challenge for the study group (Table 3).

Gender differentiated two of the ten dimensions in the study. “Physical well-being” was significantly higher in boys compared to girls (55.1 ± 17.1 points vs. 48.0 ± 14.3 points; *p* = 0.019, Mann–Whitney U test). Similarly, significantly higher results were observed in “self-perception,” where boys scored 68.6 ± 19.2 points, compared to girls’ score of 58.5 ± 22.6 points (*p* = 0.031).

The age of the participants was significantly and negatively correlated with scores in the “moods and emotions” domain (*r* = −0.216; *p* = 0.031), indicating a weak association—older age was linked to lower ratings of mood and emotions.

Similarly, age was negatively associated with “peer relationships” (*r* = −0.300; *p* = 0.002), corresponding to a weak-to-moderate association—older adolescents reported poorer peer relationships.

Participants living in rural areas scored higher in “relationships with parents and home” compared to those living in urban areas (78.7 ± 13.4 points vs. 68.2 ± 21.6 points; *p* = 0.025). However, whether children were raised in whole or single-parent families did not significantly affect the KIDSCREEN-52 scores.

Socioeconomic status significantly influenced two HRQoL variables: “relationships with parents and home” and “financial resources.” Adolescents reporting very good/good financial conditions at home scored significantly higher, with 73.1 ± 20.3 points for “relationships with parents and home” and 74.1 ± 22.5 points for “financial resources.” In contrast, those with average/poor financial conditions scored lower: 62.3 ± 17.4 points and 51.4 ± 29.0 points, respectively.

Children experiencing pain reported significantly worse “physical well-being” (42.1 ± 13.7 points vs. 53.0 ± 15.9 points; *p* = 0.026) and “moods and emotions” (56.9 ± 22.4 points vs. 69.7 ± 20.2 points; *p* = 0.041).

## 4. Discussion

This study offers valuable insights into the health-related quality of life (HRQoL) of adolescents hospitalized for cardiological conditions, reaffirming its multifactorial nature. The results emphasize that HRQoL is affected not only by clinical symptoms but also by sociodemographic and psychosocial factors.

The highest scores were observed in the “social acceptance” domain, suggesting that most adolescents in our study felt accepted by their peers. This result aligns with Ravens-Sieberer et al. [20], who reported similarly elevated scores in the social dimension among chronically ill adolescents across Europe. Ferro and Boyle [11] found that adolescents with chronic conditions who maintain strong peer relationships report better emotional well-being. Peer acceptance can act as a protective factor that buffers emotional distress, as previously reported in international studies [11,15,20].

In contrast, the “school environment” was the lowest-rated domain in our sample. This reflects findings from Caggiano et al. [19], who reported similar challenges in children with chronic illnesses the U.S., where academic setbacks and social withdrawal were often linked to frequent absences and a lack of tailored support. Latal et al. [10] also observed that Swiss children with congenital heart disease frequently face academic stress that impacts emotional functioning. These international parallels strengthen the case for integrated, systemic interventions that improve educational inclusion and provide psychosocial support.

From a clinical and nursing perspective, these results suggest that school-related difficulties deserve priority attention in the care of adolescents with cardiological conditions. Potential approaches may include collaboration between pediatric nurses, families, and schools to design individualized education plans, advocate for flexible learning environments, and promote communication between healthcare and educational institutions. While the current study does not evaluate the effectiveness of such interventions, our findings emphasize the need to address the school environment as a central component of holistic, family-centered care.

Social acceptance, again emerged as a key component of successful adaptation to illness. Prior research confirms that adolescents with chronic heart conditions benefit significantly from social support networks, which help buffer psychological stress [21,22,23]. Conversely, limited peer contact was associated with increased symptoms of anxiety and depression, echoing findings by Lawoko and Soares [24] that identified social isolation as a major predictor of reduced HRQoL in this population.

Pediatric nurses play a crucial role in reducing psychosocial distress. Initiatives such as nurse-led peer support groups, mental health screenings, and educational advocacy have been shown to enhance emotional resilience, school engagement, and overall quality of life [22,23,25]. These findings are supported by a recent scoping review by Dave et al. [15], which concluded that peer support interventions significantly improve psychological outcomes and social integration among adolescents with chronic or rare conditions. Nurse-led programs like these are effective in strengthening emotional and social well-being in young patients.

Notably, adolescents with complex congenital heart disease often experience functional limitations, emotional insecurity, and uncertainty about the future, underscoring the need for individualized nursing care [26,27].

School-based nursing programs—including chronic illness education and teacher training in cardiac emergency protocols—can improve peer inclusion and reduce stigma [28]. Repeated hospitalizations, medical restrictions, and poor coordination between healthcare and schools are major contributors to academic difficulties and social disconnection [29]. Nurses are well positioned to advocate for Individualized Education Plans (IEPs) and support successful reintegration into the academic environment [28]. Tele-education tools, including hybrid learning models and remote tutoring, may further support educational continuity for frequently absent students [19].

A limitation of this study is the heterogeneity of the patient population, which included both adolescents with complex congenital heart defects and those with more stable conditions such as controlled arrhythmias. This variability may have influenced HRQoL outcomes; however, it also reflects the real-world clinical context of pediatric cardiology wards. Future studies with larger, diagnosis-specific cohorts are needed to clarify condition-specific determinants of HRQoL.

### 4.1. Socioeconomic Disparities and HRQoL

Another critical determinant of HRQoL is socioeconomic status (SES). Adolescents from financially stable families reported better psychological and physical health, consistent with research showing that financial security facilitates treatment adherence and access to quality care [11,29]. By contrast, a lower SES is linked to delayed care, reduced adherence, and increased caregiver stress—all of which negatively affect HRQoL [30]. Poor nutrition, unstable home environments, and lack of access to rehabilitation services may further deepen disparities [31]. Nurses can address these challenges by facilitating financial counseling, connecting families with community support, and referring them to subsidized services [32]. Expanding nurse-led outreach and psychosocial support programs may mitigate these effects and promote equity in care outcomes [30].

### 4.2. Future Implications and Research Directions

Future studies should adopt longitudinal methodologies to track the long-term effects of nurse-led interventions. Key areas for further investigation include:

- Psychosocial support programs for adolescents with congenital heart disease (CHD).
- School-based nursing interventions that facilitate reintegration and improve academic success.
- Family-centered nursing models that improve parental coping and involvement in treatment.

A better understanding of which nursing interventions yield the most significant improvements in HRQoL will inform best practices in pediatric cardiac care and guide future clinical policies.

## 5. Conclusions

The health-related quality of life (HRQoL) of adolescents hospitalized for cardiac conditions is mainly affected by socio-demographic factors, such as gender, age, place of residence, perceived socioeconomic status, and experiences of pain and discomfort. Girls, older adolescents, urban residents, and those reporting poorer socioeconomic conditions and pain had a lower HRQoL scores in specific areas. Conversely, family structure and the presence of chronic diseases did not significantly influence HRQoL outcomes.

Future research should focus on multicenter, long-term studies of scalable nurse-led interventions and conduct cost-effectiveness analyses of digital tools, such as mHealth apps, designed to monitor symptoms and support treatment adherence. These approaches are vital for enhancing long-term well-being and optimizing care for this vulnerable patient group.

## Figures and Tables

**Table 1:** Sociodemographic characteristics of the study group.

Variables	Subgroup	N	%
Gender	Female	54	54%
	Male	46	46%
Age	11–14 years	58	58%
	15–18 years	42	42%
Living	City	67	67%
	Village	33	33%
Raised	Full Family	72	72%
	Single-Parent Family	28	28%
Socioeconomic conditions	Bad	2	2%
	Average	16	16%
	Good	53	53%
	Very good	29	29%

Legend: N—number, % percentage of the total sample represented by each subgroup.

**Table 2:** Reasons for hospitalization in the cardiology ward among the study participants (n = 100).

Reason for Hospitalization	N	%	Medical Diagnosis
Exacerbation of chronic cardiac disease symptoms	5	5	Cardiac arrhythmias (4 children), Pulmonary arterial hypertension (1 child)
Chronic comorbidities cooccuring in children with cardiac disease	19	19	Thyroid disorders (6), bronchial asthma (5), food and inhalant allergies (4), ulcerative colitis (2), celiac disease (1), epilepsy (1)
Monitoring after cardiac surgery for congenital heart defects	9	9	Follow-up after correction of congenital heart defects
Cardiac diagnostics	67	67	Syncope, tachyarrhythmias, bradyarrhythmias, chest pain

Legend: N—number, % percentage of the total sample represented by each subgroup.

**Table 3:** Descriptive analysis of the results for the KIDSCREEN-52 scale.

Compared Groups	N	M	SD	Min	Q1	Median	Q3	Max
Physical Well-being	100	51.5	16.0	15	40	55	60	90
Psychological Well-being	100	56.8	21.8	8	42	58	71	100
Moods and Emotions	100	67.9	20.9	11	57	71	84	100
Self-perception	100	63.5	21.5	10	55	65	80	100
Independence	100	60.9	22.8	5	45	60	80	100
Relationships with Family	100	71.1	20.2	10	58	75	86	100
Financial Resources	100	70.0	25.2	0	58	75	92	100
Social Support and Friends	100	56.0	24.9	0	35	58	79	100
School Environment	100	49.2	21.4	0	38	50	63	92
Social Acceptance	100	86.3	17.9	0	83	92	100	100

Legend: N—number, M—Mean; SD—Standard Deviation; Min—Minimum; Q1—First Quartile; Median, Q3—Third Quartile; Max—Maximum.

## Data Availability

The data presented in this study are available on request from the corresponding author.
